# The Mexican Biobank Project promotes genetic discovery, inclusive science and local capacity building

**DOI:** 10.1242/dmm.050522

**Published:** 2024-02-01

**Authors:** Mashaal Sohail, Andrés Moreno-Estrada

**Affiliations:** ^1^ Genómica Computacional, Centro de Ciencias Genómicas (CCG), Universidad Nacional Autónoma de México (UNAM), 62209 Cuernavaca, Morelos, México; ^2^ Unidad de Genómica Avanzada (UGA-LANGEBIO), Centro de Investigación y Estudios Avanzados del IPN (Cinvestav), 36821 Irapuato, Guanajuato, México

**Keywords:** Biobanks, Diversity, Genomics

## Abstract

Diversifying genotype–phenotype databases is essential to understanding complex trait and disease etiology across different environments and genetic ancestries. The rise of biobanks across the world is helping reveal the genetic and environmental architecture of multiple disease traits but the diversity they capture remains limited. To help close this gap, the Mexican Biobank (MXB) Project was recently generated, and has already revealed fine-scale genetic ancestries and demographic histories across the country, and their impact on trait-relevant genetic variation. This will help guide future genetic epidemiology and public health efforts, and has also improved polygenic prediction for several traits in Mexican populations compared with using data from other genome-wide association studies, such as the UK Biobank. The MXB illustrates the importance of transnational initiatives and funding calls that prioritize local leadership and capacity building to move towards inclusive genomic science.

## Teaser

**Summary:** The Mexican Biobank captures biological factors at a nation-wide scale for the first time in Latin America. It is an inspiring example for leading such initiatives in the Global South.

## Why diversify genotype–phenotype databases and studies?

In 2022 the International Common Disease Alliance (ICDA) published a series of recommendations for the next phase of complex-disease genetics, and recommendation number two urges researchers to: “Increase the diversity of biobanks focused on genetic analysis, by establishing and expanding biobanks in Africa, the Americas, and Asia” (see International Common Disease Alliance (IDCA) – Recommendations and White Paper). The issue is that studies attempting to understand the relationship between genotype and phenotype, predominantly genome-wide association studies (GWAS) and other epidemiological studies, have been dominated by participants of European genetic ancestries (Popejoy and Fullerton, 2016).

While there are multiple factors – historical, economic and political – for this bias, it is also due to the assumption of a shared genetic architecture of traits across diverse genetic ancestries. This assumption is particularly prevalent for drug discovery and therapeutics. However, it has been shown that genetic background, whether autosomal or mitochondrial, can be relevant for variations in drug response and other biomedical traits (Lagos et al., 2010; Moreno-Estrada et al., 2014). Recent research has also made clear that the accuracy of prediction of a trait or a complex disease by using polygenic scores decays when applied to individuals of diverse backgrounds, whose ancestries were underrepresented in the GWAS cohort (Ding et al., 2023). This is likely to be due to a combination of variation in allele frequency (Box 1), linkage disequilibrium (Box 1), causal genetic and epistatic effects (Box 1), epigenetic variation (Box 1), and gene−environment interaction (Box 1) across this ancestry continuum (Martin et al., 2019; Wang et al., 2020). Other factors, such as gender and socioeconomic status, also affect the accuracy of prediction from the GWAS cohort to the prediction cohort (Mostafavi et al., 2020).

**Box 1. Glossary**

**Allele frequency:** Frequency of DNA variants at a genetic locus, with the genetic locus being a certain position within the DNA sequence.

**Archaic introgression:** Humans also carry genetic material from Neanderthals and Denisovans as part of our genomes due to our mating history; this is commonly referred to as archaic introgression.

**Cosmopolitan:** Cosmopolitan was used to refer to individuals inferred to have mixed genetic ancestries from indigenous, European and African origins (see Moreno-Estrada et al., 2014; Romero-Hidalgo et al., 2017; Ávila-Arcos et al., 2020; Spear et al., 2020; García-Ortiz et al., 2021; Rodríguez-Rodríguez et al., 2022).

**Epigenetic variation:** Chemical modifications to a DNA sequence, which do not alter the underlying nucleotide sequence and may impact gene activity within this sequence.

**Epistatic effect:** The modifying effect (i.e. masking, inhibiting, suppressing) the expression of one or more genes has on another gene.

**Gene−environment interaction:** When environmental exposure has different effects on disease risk in individuals with different genotypes, or when individuals with the same genotype have different disease risk in different environments.

**Global South:** Nations – broadly in regions of Africa, Latin America and the Caribbean, Asia (without Israel, Japan and South Korea) and Oceania (without Australia and New Zealand) – regarded as having lower levels of economic income, newly industrialized and often former subjects of colonialism. The Global North is the other grouping, with their nations often being described as developed.

**Indigenous:** Indigenous has been used to identity and self-identify individuals and cultures who peopled Mexico and other parts of Latin America in pre-classical (2500 BC to 200 AD), classical (200 to 900 AD) and post-classical (900 to early 1500 AD) periods before the arrival of the Spanish. It is constructed in opposition to mestizo/mixed and Español/Spanish descent. In self-identity, different indigenous people identify with different cultures (Maya, Mexica, Wixarika, etc.).

**Identical by descent:** A genetic region that is identical in two or more individuals due to shared descent from a common ancestor.

**Linkage disequilibrium:** Non-random association of two genetic loci.

**Runs of homozygosity (ROH):** When a genetic segment is identical by descent in two individuals, their child will have a run of homozygosity in that genomic region, as two identical copies of that genetic segment, i.e. identical haplotypes, are inherited from each parent.

**Fig. 1.**
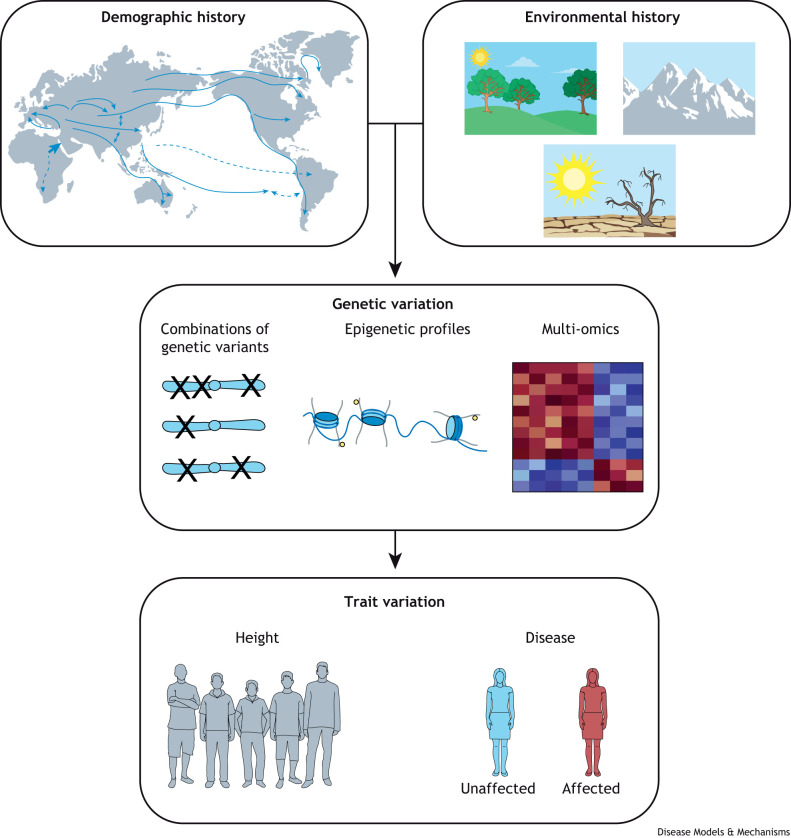
**Why diversify genotypic–phenotypic databases?** Demographic and environmental histories (usually captured by proxies of genetic ancestries in statistical analyses) impact genetic variation, such as the combination of genetic variants observed together, and epigenetic and expression profiles. These, in turn, impact patterns of trait variation. Diverse genotypic–phenotypic databases allow the identification of the most relevant genetic markers, biomarkers and gene–environment interactions for targeted therapies, and preventative medicine for all.

Therefore, even though GWAS-inferred sizes of genetic effects may be highly correlated across ancestries (Hou et al., 2023) – due to the other genetic and environmental factors described above, and as demonstrated in our recent study (Sohail et al., 2023) – understanding of trait or disease etiology and prediction is likely to be superior with increased similarity between the study and prediction cohorts. Here, by disease etiology, we mean the specific genetic and environmental factors that independently, or in interaction, contribute to trait variation or disease manifestation. It is in this overall light that diversifying genotype–phenotype databases and studies are essential for making preventative and precision medicine accessible and available to all humans who are spread across a continuum of genetic ancestries and environments (Fig. 1).

## The growth of global biobanks

**Fig. 2.**
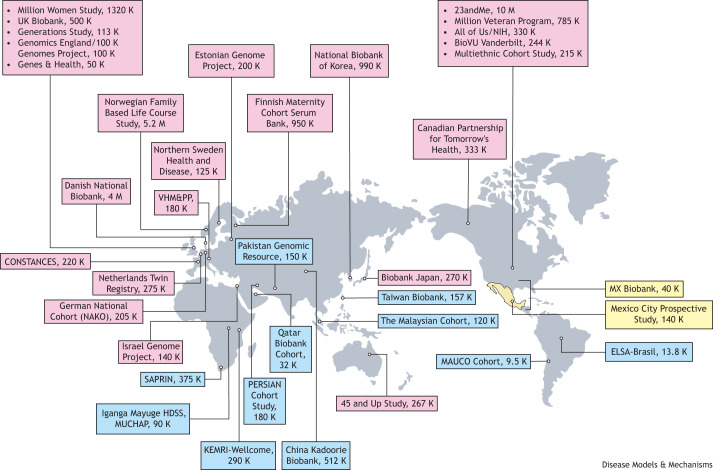
**Global biobanking.** The major biobanks included in the International Health Cohorts Consortium (IHCC), selecting the top study (i.e. the largest according to current enrollment) for each country – except for the USA and UK, for which we present the top five studies of each country. Biobanks from the Global North are presented in pink, while biobanks from the Global South are presented in blue. Biobanks from Mexico are shown in yellow, the square bracket indicating the nationwide nature of the MXB. MAUCO, Chilean Maule Cohort; NAKO, Nationale Kohorte; NSHDS, Northern Sweden Health and Disease Study; VHM&PP, Vorarlberg Health Monitoring & Promotion Programme.

Despite the overall increase in the number of participants from diverse ancestries in GWAS [see the International Health Cohorts Consortium (IHCC)] and Mills and Rahal (2020), the majority of the large-scale biobanking efforts are skewed towards cohorts recruited in countries from the Global North (Box 1), which are largely representing participants of European and, to a lesser extent, Asian ancestries. The IHCC has been cataloging the major global biobanks that aim to recruit ≥100,000 participants (see IHCC; Fig. 2). Out of 70 studies reported in the official IHCC portal as of November 14, 2023, only 19 are located in countries outside the Global North, and only eight (11%) are primarily non-Eurasian cohort studies (Fig. 2). This unveils a major gap of large-scale biobanks in underrepresented regions, such as Latin America, and prompts bigger efforts that include and benefit these communities. The proportion of GWAS participants that self-identify as Hispanic or Latin American keeps shrinking as large biobanks of European ancestries become even larger (Mills and Rahal, 2020). This is not only an issue of representation but, also, of statistical power to enable genetic discoveries and assessment of genetic predispositions that are relevant to all populations. Therefore, supporting the creation of diverse biobanks is a scientific imperative for the human genetics community.

The only two cohort studies of Latin Americans outside of Mexico included in the IHCC catalog are the Brazilian Longitudinal Study of Adult Health [i.e. Estudo Longitudinal de Saúde do Adulto (ELSA)-Brasil cohort (Schmidt et al., 2015)] and the Chilean Maule Cohort (MAUCO) studies (Ferreccio et al., 2020). The former is a cohort from five urban areas in Brazil, while the latter – despite not having yet reached the 100,000 participants size – is a pioneering example of large-scale prospective recruitment with biobanking standards conducted in Latin America and will follow up with participants over 10 years. However, the MAUCO study is restricted to participants resident in a single region of southern Chile – the Maule region. Similarly, the Mexico City Prospective Study (MCPS), despite its larger size (140,000 participants), is restricted to participants recruited in two adjacent borrows within Mexico City (Coyoacán and Iztapalapa), and its biobank is not maintained within Mexico but at the University of Oxford (UK) (Ziyatdinov et al., 2023). Therefore, none of these cohorts have nationwide sampling coverage, which limits the ability to assess a broader array of ancestries and environments that are part of these Latin American countries. These limitations motivated the launching of complementary efforts – incorporating broader sampling across Mexico and local biobanking – which resulted in the Mexican Biobank (MXB) Project, the first nationwide genomic characterization of a major country in Latin America.

When we talk about genomic biobanks and DNA collections, we rarely think about the local institutions, communities and participants behind these monumental efforts, […]

## Opportunities and challenges in the creation of the MXB

When we talk about genomic biobanks and DNA collections, we rarely think about the local institutions, communities and participants behind these monumental efforts, nor the challenges involved in conducting competitive science in low- and middle-income countries (LMICs). Despite the consensus on diversifying biobanks around the world, most of the existing biobanks with diverse ancestries have conducted recruitment in the Global North, thus not capturing the rich continuum of ancestries and environmental exposures of the Global South (Box 1). Others have partnered with local institutions to conduct recruitments in the Global South but most of the remainder of the research in these studies takes place abroad, which limits the potential for strengthening the in-country capacity to lead scientific research. Unfortunately, this has been a common collaborative scheme over several years, which is more prone to models of helicopter science (see https://www.statnews.com/2022/08/11/oxford-mexican-research-team-diversify-genomic-databases/). To overcome this, the MXB leadership – headed by one of the authors (A.M.-E.) and by Dr Lourdes García-García of the Instituto Nacional de Salud Pública (INSP) – envisioned the MXB Project with a different perspective: one that promotes local leadership and local capacity building. Cinvestav partnered with Mexico's INSP that has conducted National Health Surveys since 1988, to collect nutritional, demographic and biomedical data, together with biological samples from all over the country. National Health Surveys (originally conceived as National Nutrition Surveys) are conducted periodically, so the community is engaged with the study and receptive to household visits by INSP staff and fieldwork teams. The MXB is based on the National Health Survey designed by Dr Jaime Sepúlveda and conducted in the year 2000 (Sepúlveda et al., 2007), which involved a two-hour visit to each household and collected more than 40,000 blood samples from adult donors. Prior to recruitment, the team met with the political, religious and community leaders of each locality to communicate the nature of the study, answer all questions and engage with the community. This community engagement process was essential at every recruitment site, with an emphasis on ensuring indigenous and rural communities understood their participation in the study.

Before the MXB, these valuable collections had only been partially characterized genetically, and no genome-wide assessment had been conducted in a nationwide cohort, mostly due to insufficient resources to generate genomic data and lack of the local infrastructure to process and analyze the samples. To tackle this, A.M.-E. and Lourdes García-García had a clear roadmap for seeking funds: scale up the medical genomics research capacity in Mexico by locally generating data, recruiting and retaining talented scientists, and increasing international competitiveness of local institutions. After a first round of funding, jointly supported by the Consejo Nacional de Humanidades, Ciencias y Tecnologías (National Council of Humanities Science and Technology, CONAHCYT) and the Newton Fund from the UK, the MXB was able to install the capacity to genotype and analyze DNA samples locally at the Advanced Genomics Unit (UGA-LANGEBIO) of Cinvestav in Irapuato, Mexico. Such decisive transnational funding was key to unlocking the potential of the biobank, as there are only limited funds available locally for large scientific projects. This, unfortunately, is the case in many LMICs across Latin America and will continue to be the trend unless local governments give science higher priority.

The MXB genotyped more than 6000 DNA samples, using microarrays to analyze 1.8 million single nucleotide polymorphisms (SNPs) distributed across the genome per individual. This technical task alone was a major challenge due to the unprecedented scale of effort for the existing local capacity and other adverse circumstances associated with conducting genomics research in Latin America, such as increased costs of reagents due to importation taxes and distributor's markup, and limited availability of specialized staff. These challenges were overcome by several initiatives that leveraged the unique position of UGA-LANGEBIO as a national reference in Mexico for genomic research. A.M.-E., who was in charge of the genome core facility, partnered with microarray manufacturer Illumina, Inc. to secure heavily discounted pricing for reagents used for genotyping by the MXB, so that the core facility could become a competitive alternative not only for this project but for others throughout Latin America. Additionally, the core facility and its staff had experience in DNA sequencing, and their capacity was ready to be scaled-up for processing a larger volume of samples. Therefore, funding from the MXB allowed for an automated DNA prepping system to be installed, technicians to be trained and students to be taught computational skills to analyze large genomic datasets, which usually represents another major challenge for the region. As a result, the genotyping success rate of the MXB was >99.9% (fewer than 60 samples failed genotyping). Furthermore, sophisticated data analyses were implemented by putting together a transnational team of experts across participating institutions in Mexico, the US and the UK, with expertise in genetic epidemiology, population genetics and complex trait genetics to enable a high standard and rigor for the analyses performed. This collaborative network enabled joint analyses by promoting data sharing and common access to available servers, which also allowed for the computational burden to be shared.

Under this collaborative scheme, the data were analyzed by the local team in Mexico with input from the international consortium while maintaining local leadership of the project as this was the MXB vision from the outset. This is not a trivial point since many Global South countries, despite moderate limitations, are able to generate good quality data but often end up handing the data analysis phase to foreign groups because of bioinformatic limitations, a step that compromises the training potential for local researchers and the transmissibility of knowledge to next generations. Fortunately, a project of this caliber is able to generate positive traction in the reverse direction as it is attractive enough to recruit international talents and contribute to local training. This was the case for M.S., who joined the project as a postdoctoral fellow with A.M.-E. at UGA-Langebio, Cinvestav and is now an Associate Professor at the Universidad Nacional Autónoma de México (UNAM). M.S., who led the analyses of the MXB data at Cinvestav and, later, at UNAM in Mexico, also spent time during her postdoctoral training at the INSP, the University of Chicago (IL, USA) and University of Oxford (UK) to integrate the expertise of leaders in global medical genetics and population genetics, such as Drs Alexander Mentzer and Adrian Hill (The Wellcome Centre for Human Genetics, Oxford, UK) and Dr John Novembre (Department of Human Genetics, University of Chicago, IL, USA). These cooperative exchanges also propagated knowledge and skills to MXB trainees across multiple institutions and will be reflected in stronger future generations of genomic scientists. Besides our recent flagship study (Sohail et al., 2023), at least two other publications have used data from the MXB (Medina-Muñoz et al., 2023; Jiménez-Kaufmann et al., 2022) and many more are ongoing, giving more students and collaborators the opportunity to be trained in computational tools and to answer different research questions. The data are hosted at the European Genome-phenome Archive (EGA) and accessible to the broader research community by submitting a research proposal to the MXB Data Access Committee (DAC). Given the sample size of the MXB, there is huge potential for scaling up and accelerating genetic discoveries by characterizing the full set of 40,000 samples.

With the goal of diversifying genomics to enable a more-complete and accurate science, and a global spread of the benefits, the MXB is an illustrative example. In this Perspective, we identified factors that are essential for the future of human genetics and genomics in order to build inclusive science and scientific workforces:

(1) Tapping into existing biobanks that hold data collected by local institutes of public health and similar establishments.
(2) Transnational funding calls that allow sharing of resources across economies with the goal of developing and strengthening locally based scientific initiatives, prioritizing those in LMICs to reduce inequities.
(3) Diversifying the genomics workforce by training local scientists, while creating international teams that share expertise in fruitful reciprocal collaborations.
(4) Allowing trainees to move transnationally to gain necessary experience and expertise.
(5) Databases, methods and data interpretation that jointly incorporate genetic and environmental factors, as well as ancestries at multiple timescales.

This flagship study will help to inform future genetic and epidemiological work as well as public health interventions.

## What have we learned and what we hope to learn

Through our flagship analysis of the MXB, we built on previous work studying genetic data from ‘indigenous’ (Box 1) and ‘cosmopolitan’ (Box 1) groups across Mexico (Moreno-Estrada et al., 2014; Romero-Hidalgo et al., 2017; Ávila-Arcos et al., 2020; Spear et al., 2020; García-Ortiz et al., 2021; Rodríguez-Rodríguez et al., 2022). With the nationwide sampling strategy of the MXB, we observed a genetic structure that – most clearly – separates the indigenous Mayan region from the other cultural regions, and highlights the genetic diversity present within this and other cultural regions. We observed evidence of varying historical trajectories of populations in different cultural regions of Mexico, as inferred from genetic segments that are identical by descent (Box 1). This highlighted different indigenous civilization histories, as well as colonial and post-colonial histories that also involved individuals from Europe and Africa. In particular, we observed populations inferred from ‘indigenous’ genetic segments to be growing in size within cultural regions – such as the State of Oaxaca and the Mayan region – more than in the centre of Mexico. This highlights that the European colonization and its subsequent impact on diminishing indigenous groups was heterogeneous in different parts of Mexico.

As described above, in the MXB we considered individuals and their genomes at several geographic resolutions that reflect different timescales for the purposes of population grouping. The first level of grouping we used is that of the Mesoamerican regions, reflecting indigenous cultural histories (Box 1). The second level is that of continental ancestries to characterize the genomes according to such roots, given that they came together in the colonial and post-colonial periods in the Mexican populations. Last, we also considered an axis of ancestry variation within indigenous ancestries. This axis captures a wide range of genetic variations found between indigenous groups from within south-eastern Mexico in the Mayan region to the northern areas near Mexico's border with the US. Such multi-resolution grouping was conducted in this study (Sohail et al., 2023) in an effort to embrace a multi-dimensional continuous view of ancestry.

We also observed genome patterns probably shaped by demographic events, such as the founder effect of the small groups who crossed the Bering Strait to first populate the Americas. We observed, for example, that segments of the genome with genetic ancestries from the Americas tend to be found more often in short runs of homozygosity (ROH) (Box 1). This is relevant for diseases that have a recessive or partially recessive architecture, i.e. where the disease is only manifested when both copies of a genetic allele are present – as is the case at a homozygous genetic locus. Further, we observed fewer rare genetic variants in these genetic segments compared to those with ancestries from Europe, Africa or Asia in Mexico. This is because founder effects can lead to rare variants being lost or driven to higher frequencies; such founder effects are more dominant in indigenous genomes due to the Bering Strait bottleneck and later history of maintaining small group sizes.

With respect to complex traits, we observed that genetic ancestries from the Americas are associated with shorter height, higher levels of glucose and triglycerides, and lower levels of creatinine and low-density lipids. On the one hand, we did not observe an association between genetic ancestries and body mass index (BMI), levels of high-density lipids, total cholesterol and blood pressure. On the other hand, although BMI showed no association with genetic ancestries, a high proportion of an individual's genome being in ROH is associated with lower BMI, whereas living in an urban environment is associated with higher BMI. This exemplifies the conjunction of genetic and environmental factors in complex traits.

When looking at genetic associations to disease and overall disease predisposition, we found that we, indeed, predict several traits and diseases in Mexicans better or similarly well when using our MXB genetic associations compared to those obtained through the much larger UK Biobank GWAS. This included triglyceride, glucose, creatinine and cholesterol levels. However, we did not find any novel genetic SNP associations that are significant only in the MXB and have not been reported before. This may change with a larger sample size, as some novel associations have been suggested in our analysis.

While the Mexico City Prospective Study (MCPS) (Ziyatdinov et al., 2023) is larger in size, the MXB Project has national coverage across rural and urban localities in all 32 states of Mexico, serving as a reference dataset for Mexico with unprecedented resolution. Like other major cosmopolitan urban areas, Mexico City, indeed, harbors abundant diversity, although this is predominantly from nearby regions, mainly central and southern Mexico. In contrast, Fig.1 makes clear what is gained in terms of genetic diversity when a sampling approach like that adopted in the MXB Project is taken. Different regions of Mexico represent the diversity within and amongst them, and this is only possible through such rich diverse sampling. Both the MCPS and the MXB project are valuable and complimentary resources, promising a bright future for Mexican genomics research and for the development of precision medicine in the country.

The MXB Project flagship study will help to inform future genetic and epidemiological work as well as public health interventions. Within our own collaborative network, we are now using the MXB database to study variations in maternal and paternal lineages in Mexican histories, the association of mitochondrial variations with trait variations and disease predispositions, the correlation of inferred genetic effects on traits across different genetic ancestries, and the distribution of clinically relevant genetic variation for Mendelian traits across Mexico. The database is also being used for genetic association analysis of metabolic diseases, combining the MXB Project with other disease cohorts. We are also conducting further work to understand how best to predict trait or disease predisposition in Mexico, and the role of archaic introgression (Box 1) in trait variation and disease predisposition.

[…] lead locally, collaborate globally.

## Perspectives for future work

It took some hopeful scientists putting together a team and seeking funds that gave root to this project. Their vision was important: lead locally, collaborate globally. It is simply a matter of perspective, to believe and demonstrate that there is absolutely nothing preventing such large-scale population and medical genomics databanks to be generated and analyzed locally in the Global South. This perspective has to be shared globally and locally. As several historical and global factors have left many countries in the Global South at a disadvantage, fruitful reciprocal collaborations as well as transnational funding sources are key for progress in the short and intermediate term. With the data generated, much research can now proceed with this resource with limited additional cost. By training local leaders and building local capacity, the research is locally sustainable and generates a life of its own. This is the inspiration we hope the MXB Project can provide to other such aspiring projects and leaders in the Global South and North alike. The MXB Project and similar initiatives will allow disease predisposition profiles to be analyzed with best precision by using biobanks closely related to the local groups at risk, which will lead to succeeding benefits in preventative and precision medicine to be widely available.
